# multiclassPairs: an R package to train multiclass pair-based classifier

**DOI:** 10.1093/bioinformatics/btab088

**Published:** 2021-02-05

**Authors:** Nour-Al-Dain Marzouka, Pontus Eriksson

**Affiliations:** Department of Clinical Sciences, Division of Oncology, Lund University, 22381 Lund, Sweden; Department of Clinical Sciences, Division of Oncology, Lund University, 22381 Lund, Sweden

## Abstract

**Motivation:**

k–Top Scoring Pairs (kTSP) algorithms utilize in-sample gene expression feature pair rules for class prediction, and have demonstrated excellent performance and robustness. The available packages and tools primarily focus on binary prediction (i.e. two classes). However, many real-world classification problems e.g. tumor subtype prediction, are multiclass tasks.

**Results:**

Here, we present multiclassPairs, an R package to train pair-based single sample classifiers for multiclass problems. multiclassPairs offers two main methods to build multiclass prediction models, either using a one-versus-rest kTSP scheme or through a novel pair-based Random Forest approach. The package also provides options for dealing with class imbalances, multiplatform training, missing features in test data and visualization of training and test results.

**Availability and implementation:**

‘multiclassPairs’ package is available on CRAN servers and GitHub: https://github.com/NourMarzouka/multiclassPairs.

**Supplementary information:**

Supplementary data are available at *Bioinformatics* online.

## 1 Introduction

Single sample predictors have the benefit that in-sample measurements and relationships are used; consequently, a given sample is classified in an absolute manner avoiding the need for normalization toward a reference cohort. k-Top-Scoring Pairs (kTSP, Tan *et al.*, 2005) single sample predictors have demonstrated good performance across platforms (Cirenajwis *et al.*, 2020; Paquet and Hallett, 2015; Tan *et al.*, 2005; Xu *et al.*, 2005). kTSP uses a majority voting of a set of binary rules e.g. if **GeneA**<**GeneB**, then **Class1**, else **Class2**, to predict one of two classes. Different implementations of binary kTSP classifiers are currently available through R packages, e.g. MetaKTSP (Kim *et al.*, 2016), switchBox (Afsari*et al.*, 2015), ktspair (Damond, 2011), Rgtsp (Popovici*et al.*, 2011) and tspair (Leek, 2009). However, many real-world classification tasks are multiclass problems. Thus, to extend the rule-based approach to multiclass prediction, Tan *et al.* (2005) suggested a prediction scheme based on the voting of one-versus-rest or one-versus-one rules or using binary rules in a hierarchical approach. Popovici*et al.* (2011) suggested a decision tree-like structure in the R package Rgtsp, which is currently the only multiclass prediction approach available as an R package. Paquet and Hallett (2015) combined one-versus-rest rules using a Naive Bayes model to solve a multiclass problem. However, none of the methods outperforms all others in every aspect, and can differ in performance, computational demand and interpretability.

Here, we present the R package ‘multiclassPairs’, which provides a streamlined way to train and apply pair-based multiclass predictors using either a novel Random Forest (RF) approach or through ensembled one-versus-rest kTSP classifiers generated by the switchBox package. multiclassPairs provides additional gene and rule selection methods tailored for multiclass and multiplatform problems.

## 2 Materials and methods

To build a pair-based multiclass predictor, multiclassPairs provides two methods, either an RF-based workflow or a one-versus-rest kTSP workflow. Both have the following steps: reading the input data and labels, selecting informative features, combining features as pairs, selecting informative pairs and constructing the final predictor model. After reading the input data and labels by *ReadData* function, each workflow handles the subsequent steps differently, as described below and in further detail in the Supplementary File S1.

## 3 Random Forest scheme

multiclassPairs uses the RF algorithm which is capable of handling complex prediction tasks and can be used for feature selection. The fast RF implementation from the ranger package (Wright and Ziegler, 2017) is used in all RF steps. This includes ranking of genes (*sort_genes_RF* function) and rules (*sort_rules_RF* function) and the training of the final RF model (*train_RF* function). To deal with class size imbalance, gene and rule selection are performed using both overall and class-versus-rest importance which can be applied separately to each platform/study to select features important in all platforms/studies. The *train_RF* function optionally filters the less informative rules via the Boruta package (Kursa and Rudnicki, 2010) before building the final trained RF model. The user can determine how many times genes are allowed to be used among the rules, by default genes are not repeated (disjointed rules). The *optimize_RF* function can be used to perform parameter tuning for the final RF model. To handle potential missing values/genes in the test data, a kNN-imputation strategy has been incorporated into the prediction function (*predict_RF*).

## 4 One-versus-rest scheme

In the one-versus-rest scheme, one-versus-rest binary classifiers for each class are assembled in one model. A benefit of the one-versus-rest approach is the intuitive model interpretability. The workflow in this scheme starts with gene filtering (*filter_genes_TSP* function) which can be performed using one-versus-rest Wilcoxon test or one-versus-one Dunn’s test. The *train_one_vs_rest_TSP* function combines the filtered genes into binary one-versus-rest rules and gives a score for each rule in each class, after which an optimal number of top-scoring rules for each class is selected and assembled into a final model. Rule scores can be obtained as one-versus-rest score or as the average of one-versus-one scores. The switchBox package is used for calculating the rule scores and to determine the optimal number of rules for each class through the Variance Optimization (VO) approach (Afsari *et al.*, 2014), which is faster than the slower cross-validation approach. The user can specify a search range for candidate number of rules and allow gene repetition in rules or not. Non-variant genes (known as pivot genes), can be included in rule formation to include more possible pairs. For prediction, predict_one_vs_rest_TSP function uses either class votes i.e. number of *true* rules divided by number of rules for that class, or weighted class votes i.e. sum of the scores for the *true* rules divided by sum of rule scores for that class. Weighted votes are used by default to reduce the chance of ties. Vote ties are flagged and reported to the user. Similar to the RF workflow, gene filtering and rule scoring can be performed in a platform/study-wise manner.

## 5 Visualization

**Fig.1.**
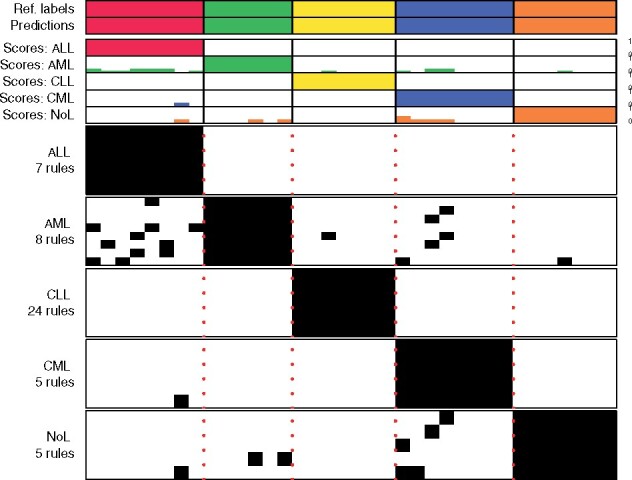
Example plot of binary rules in a leukemia training dataset. Columns represent samples. Upper panel shows the reference and predicted labels. Middle panel shows bar plots for the prediction scores. Lower panel shows the binary rules in the one-versus-rest classifiers. Black indicates ‘true’ rules and white are ‘false’

multiclassPairs provides heatmap plots to visualize the binary rules and prediction scores in the training and test datasets (Fig.1). For the RF models, a proximity matrix based on out of bag predictions can be visualized to show the class cohesiveness among the training samples.

## 6 Comparison

We used a breast cancer gene expression dataset (*n* = 3134 samples, Brueffer *et al.*, 2018) to compare the subtype prediction performance between multiclassPairs and the Rgtsp decision tree (DT) approach (Supplementary File S1). We found that the multiclassPairs outperformed DT in accuracy and training time regardless of the training dataset size. One-versus-rest and RF schemes showed similar accuracies. However, with larger training datasets RF outperformed one-versus-rest approach.

## 7 Conclusion

Here, we introduce the R package multiclassPairs, enabling easy training and application of pair-based multiclass single-sample predictors using the established one-versus-rest kTSP scheme or an RF scheme. multiclassPairs is equipped with options to handle multiclass and multi-platform scenarios.

## Supplementary Material

btab088_Supplementary_Data
