# Supplementary material for: multiclassPairs: an R package to train multiclass pair-based classifier
Source: Bioinformatics. 2021 Feb 5;37(18):3043–4. doi: 10.1093/bioinformatics/btab088 (PMC8479681; doi:10.1093/bioinformatics/btab088)
Supplement: btab088_Supplementary_Data [file btab088_supplementary_data.pdf]

Supplement: multiclassPairs: An R package to train multiclass pair-based classifier

Nour-al-dain Marzouka[1] and Pontus Eriksson[1]
[1]Department of Clinical Sciences, Division of Oncology, Lund University, Sweden

Created: 27/01/2021

Contents

1 Installation **2**

2 Workflow **2**

3 Create data object **2**

4 One-vs-rest scheme **5**
4.1 Gene filtering 5
4.2 Model training 7
4.3 Prediction 8
4.4 Visualization 11

5 Random Forest scheme **14**
5.1 Gene sorting 14
5.2 Rule sorting 15
5.3 Model training 16
5.4 Training Accuracy 17
5.5 Prediction 18
5.6 Visualization 19

6 Disjoint rules **21**

7 Comparison **21**

8 References **24**

9 Session info **25**

The R package `multiclassPairs` enables easy training and application of pair-based multiclass single-sample predictors using the established one-vs-rest kTSP scheme (Tan et al., 2005) or the novel pair-based RF scheme. `multiclassPairs` is equipped with options to handle multiclass and multi-platform scenarios.

In this supplementary file, we highlight the main functions and options in `multiclassPairs` package (v0.4.1), including training a pair-based classifier, predicting classes in test data, and visualizing the prediction scores and rules.

Also, we present a comparison between the performance of the two schemes in `multiclassPairs` and the R package Rgtsp (Popovici et al., 2011) that uses a decision tree-like structure which is currently the only multiclass prediction approach available as an R package.

1 Installation

`multiclassPairs` package is available on CRAN and GitHub. You can use the following code to install `multiclassPairs` package from CRAN and GitHub and its dependencies from Bioconductor.

```
# Install the released version from CRAN using
if (!requireNamespace("multiclassPairs", quietly = TRUE)) {
    install.packages("multiclassPairs")
}

# Or install the dev version from GitHub using
if (!requireNamespace("multiclassPairs", quietly = TRUE)) {
    if (!requireNamespace("devtools", quietly = TRUE)) {
        install.packages("devtools")
    }
    library(devtools)  # this package is needed to install from GitHub
    install_github("NourMarzouka/multiclassPairs", build_vignettes = TRUE)
}

# Install the dependencies from Bioconductor BiocManager, Biobase, and switchBox
# packages from Bioconductor are needed
if (!requireNamespace("BiocManager", quietly = TRUE)) {
    install.packages("BiocManager")
}
if (!requireNamespace("Biobase", quietly = TRUE)) {
    BiocManager::install("Biobase")
}
if (!requireNamespace("switchBox", quietly = TRUE)) {
    BiocManager::install("switchBox")
}
```

2 Workflow

The workflow in `multiclassPairs` package starts with the `ReadData` function. Two schemes are available to train the pair-based classifier:

- First option: a one-vs-rest scheme that assemble one-vs-rest binary classifiers built by `switchBox` package (Afsari et al. 2014) which uses the Top-score pairs (TSP) algorithm.
- Second option: a novel pair-based implementation of the random forest (RF) algorithm.

Both methods have the following steps: reading input data and labels, selecting informative features, combining features as pairs (i.e. rule1: feature1 < feature2, rule2: feature1 < feature3, etc…), selecting informative pairs, and constructing the final predictor model. After reading the input data and labels by `ReadData` function, each workflow performs and handles the next steps in a different way as described below. The workflow in `multiclassPairs` is summarized in the figure 1 in this document.

3 Create data object

`ReadData` takes the data (data.frame/matrix/ExpressionSet) and class labels for the samples, then generates a data object to be used in the downstream steps, such as gene filtering, training, and visualization.

Optionally, `ReadData` accepts additional platform/study labels and includes it in the data object when more than one platform/study are involved, this helps in performing the downstream steps in a platform/study wise manner where the filtering of genes/rules is performed for each platform/study separately, after which the top genes/rules across platforms/studies are selected.

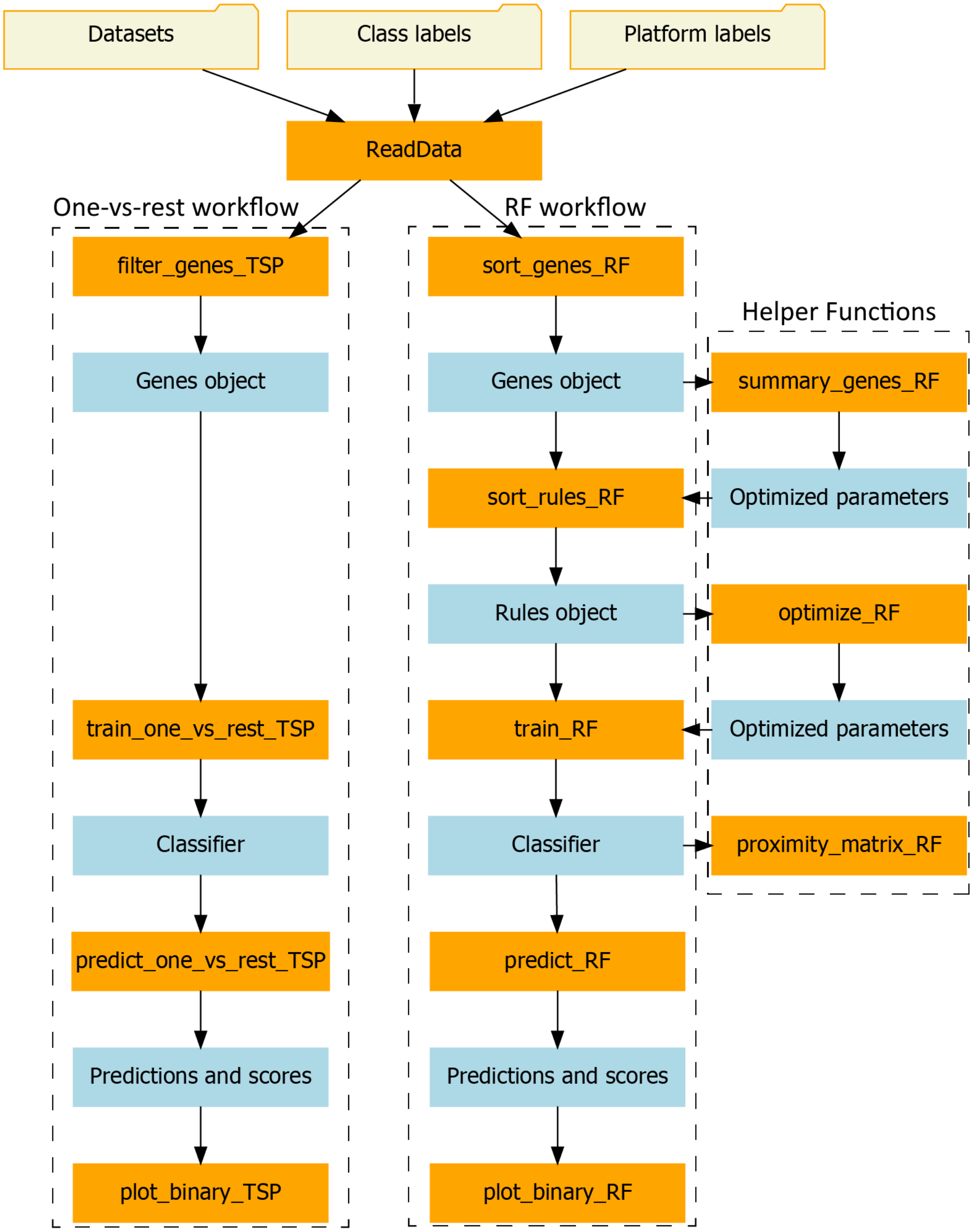

Figure 1: Workflow in multiclassPairs R package. The workflow always starts with the ‘ReadData‘ function. Then two seperated schemes are available to train pair-based classifier. First option is a one-vs-rest scheme that assemble one-vs-rest binary classifiers built by ‘switchBox‘ package which uses k Top-Score Pairs (kTSP) algorithm. The second scheme utilizes the random forest (RF) algorithm. Helper functions are optional methods to optimize the RF workflow. Inputs are colored in yellow. Functions are colored in orange. Outputs are colored in blue.

Breast Cancer data: In this supplementary file, we will use breast cancer (BC) gene expression data for samples (3134 samples and 18213 genes) (`GSE96058, Brueffer et al., 2018`). We randomly selected 1500 samples as training data and 1634 samples as test samples. We used the molecular subtypes (LumB, LumA, Basal, Her2, Normal) as labels.

```
# load multiclassPairs library
library(multiclassPairs)

# load gene expressino data
load("BC_GSE96058.Rdata")

# check the data
dim(training_data)
```

```
## [1] 18213  1500
```

```
dim(testing_data)
```

```
## [1] 18213  1634
```

```
# check the labels
knitr::kable(table(Train_Classes))
```

Train_Classes	Freq
Basal	156
Her2	154
LumA	766
LumB	324
Normal	100

```
knitr::kable(table(Test_Classes))
```

Test_Classes	Freq
Basal	170
Her2	163
LumA	817
LumB	373
Normal	111

```
# create the data object
object <- ReadData(Data = training_data, Labels = Train_Classes, Platform = NULL)
object
```

```
## multiclassPairs object
## *Data:
##    Number of samples: 1500
##    Number of genes/features: 18213
## *Labels and Platforms:
##    Classes: LumB Basal LumA Her2 Normal
##    Platforms/studies: NULL
## *Samples count table:
##  Basal   Her2   LumA   LumB Normal
##    156    154    766    324    100
```

4 One-vs-rest scheme

The one-vs-rest scheme is composed of individual binary classifiers for each class (i.e. each class versus others, Figure 2). Each binary classifier votes (i.e. gives a score) for a given sample, and the sample class is predicted based on the highest score.

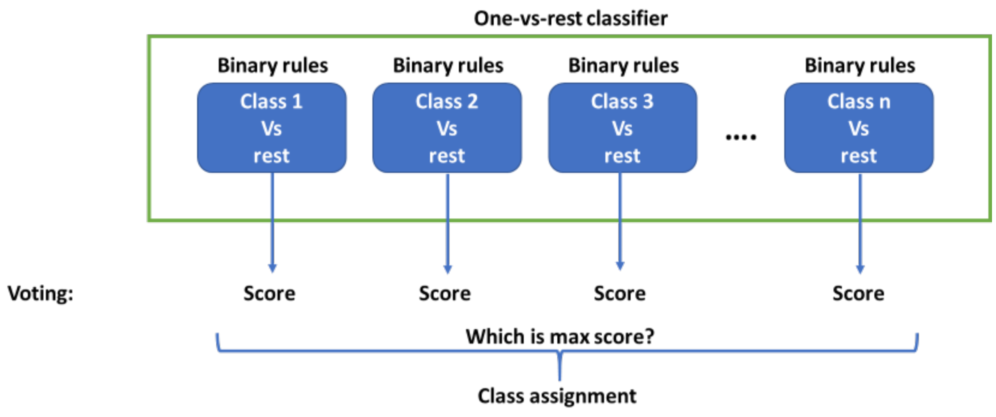

Figure 2: One-vs-rest scheme

4.1 Gene filtering

To train a pair-based classifier with the one-vs-rest scheme, we start by selecting top differentially expressed genes using the `filter_genes_TSP` function. This reduces the number of gene combinations (rules) in the next steps. This function can perform filtering in different ways to return top differentially expressed genes for each class.

`filter_genes_TSP` function provides two options for gene filtering (Figure 3). Both options begins by ranking the data (i.e. in-sample ranking). The first option performs one-vs-rest comparison using Wilcoxon test and selects a number of top up/down genes. Wilcoxon test is performed separately for each class. The second option performs one-vs-one comparisons using Dunn's test to select the top up/down genes. Dunn's test is performed for all classes together. Gene selection can be done for each platform/study separately, after which top genes across platforms/studies will be selected (Figure 4). By using one-vs-one gene filtering, more weight will be given to smaller classes. By using platform-wise gene filtering, more weight will be given to the platforms with smaller sample size. However, one-vs-one and platform-wise options do not guarantee better results but they are valid options to be considered during the training process. More details about the filtering process is mentioned in the documentation for the `filter_genes_TSP` function. The `featureNo` argument determines how many top genes will be returned for each class after filtering.

Here we will run gene filtering for the BC data.

```
filtered_genes <- filter_genes_TSP(data_object = object,
                                   filter = "one_vs_rest", # Wilcoxon test
                                   platform_wise = FALSE,
                                   featureNo = 1000, # Top genes for each class
                                   UpDown = TRUE) # 500 Up and 500 down genes
```

```
# sorted genes for One-vs-rest Scheme:
#  Object contains:
#      - filtered_genes
#         - class: LumB : 1001 genes
#         - class: Basal : 1001 genes
#         - class: LumA : 1001 genes
#         - class: Her2 : 1001 genes
#         - class: Normal : 1001 genes
```

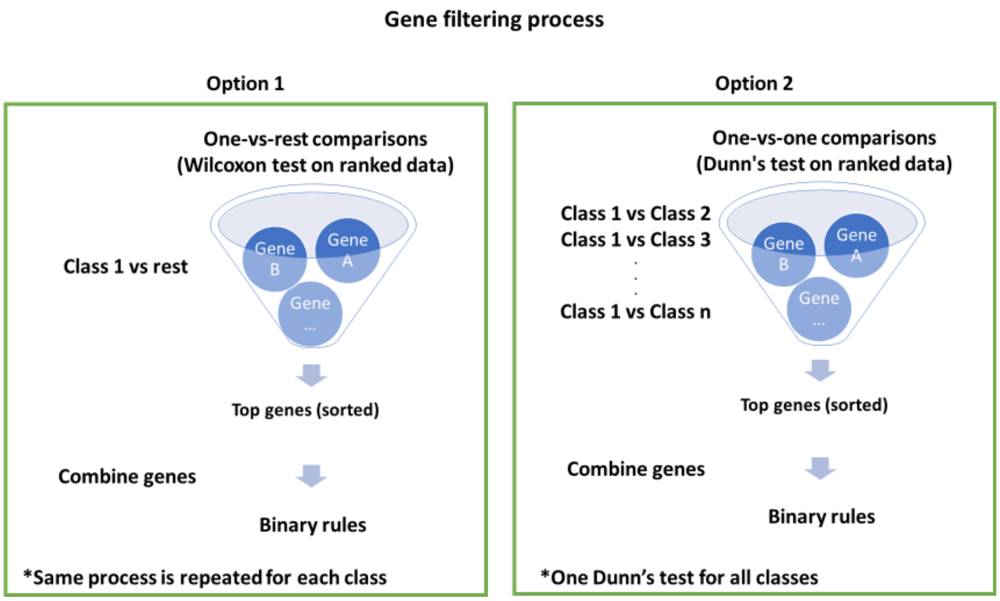

Figure 3: Gene filtering options in filter_genes_TSP function

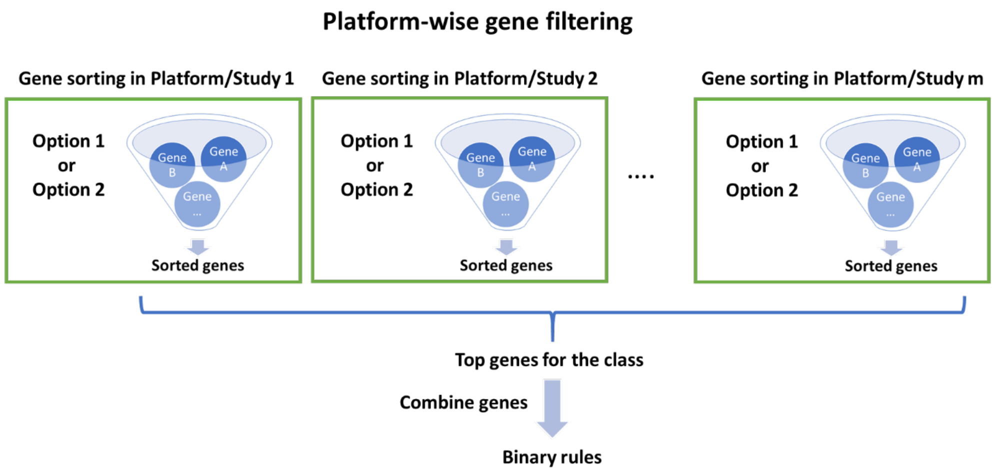

Figure 4: Platform-wise gene filtering in filter_genes_TSP function

To skip the step of filtering genes, one can create the filtering genes object with all available genes as in the next chunk. However, this significantly increases the training time.

```
# using the object that is generated by ReadData we can create genes object with
# all genes to skip filtering step

# Get the class names
classes <- unique(object$data$Labels)

# create empty genes object
genes_all <- list(OnevsrestScheme = list(filtered_genes = NULL, calls = c()))
class(genes_all) <- "OnevsrestScheme_genes_TSP"

# prepare the slots for each class
tmp <- vector("list", length(classes))
names(tmp) <- classes

genes_all$OnevsrestScheme$filtered_genes <- tmp
genes_all$OnevsrestScheme$calls <- c()
genes_all

# fill the gene object in each slot
for (i in classes) {
    genes_all$OnevsrestScheme$filtered_genes[[i]] <- rownames(object$data$Data)
}

# This is the gene object with all genes
genes_all
```

4.2 Model training

After filtering the genes we train our model using `train_one_vs_rest_TSP` function. This function combines the filtered genes as binary rules (GeneA < GeneB, GeneA < GeneC, etc.). Rules are sorted based on their score, and the best performing number of rules within the input range (i.e. `k_range` argument) is selected. The optimal number of rules is determined internally through the `SWAP.Train.KTSP` function from `switchBox` which uses the Variance Optimization (VO) method as in (Afsari et al. 2014).

Rule scoring is performed by the traditional scoring method (Geman et al. 2004). Briefly, the score for a given rule equals the percentage of samples that are TRUE for the rule in the class minus the percentage of the samples that are TRUE for the rule in another group(s). The `train_one_vs_rest_TSP` function gives two scoring options: one-vs-rest and one-vs-one. With the default one-vs-rest scoring, all the rest classes are grouped in one 'rest' group. With one-vs-one scoring option, the score is calculated as the mean of one-vs-one rule scores. The reason for using one-vs-one option is to give more weight to small classes.

Scores can also be calculated in a platform-wise manner where the score is calculated for each platform/study separately, then the mean of these scores will be used as the final score for the rule.

The `k_range` argument defines the candidate number of top rules from which the algorithm chooses to build the binary classifier for each class. Note that, during prediction process (i.e. class voting), binary classifiers with low number of rules tie more often than binary classifiers with higher number of rules. Because of that, it is recommended to use sufficient number of rules to reduce tied scores. Sufficient number of rules can depend on both the number of classes and how distinct the classes from each other.

Rules have two sides (i.e. GeneA < GeneB), usually rules constructed with two differently expressed genes (i.e. filtered genes). However, invariant genes (called pivot genes) can be involved in the rule search through `include_pivot` argument. If `include_pivot` argument is TRUE, then the filtered genes will also be combined

with all genes in the dataset, allowing rules in form of 'filtered genes < pivot gene' and 'pivot gene < filtered genes'. This increases the training time due to the increased number of possible rules.

```
# Let's train our one_vs_rest model
classifier <- train_one_vs_rest_TSP(data_object = object,
                                    filtered_genes = filtered_genes,
                                    k_range = 10:50,
                                    include_pivot = FALSE,
                                    one_vs_one_scores = FALSE,
                                    platform_wise_scores = FALSE,
                                    seed = 1234, # for reproducibility
                                    verbose = FALSE)
```

```
classifier
```

```
## multiclassPairs - One vs rest Scheme
## *Classifier:
##   contains binary classifiers:
##      - Class: LumB ... 29 rules
##      - Class: Basal ... 14 rules
##      - Class: LumA ... 25 rules
##      - Class: Her2 ... 17 rules
##      - Class: Normal ... 15 rules
```

In this trained model we now have 5 binary classifiers for the classes: LumB, Basal, LumA, Her2, Normal, with 29,14,25,17,15 rules, respectively.

4.3 Prediction

Now, let's apply our trained model on the training and test data to assess its performance. We can do this through `predict_one_vs_rest_TSP` function.

Note: If the test dataset is missing some features or genes, `predict_one_vs_rest_TSP` can deal with this by ignoring the rules with missed genes. However, if a lot of rules are absent the prediction accuracy may be affected.

`predict_one_vs_rest_TSP` returns a data.frame with class scores, score tie flags, and a final prediction based on the max score.

4.3.1 Score ties

In binary problems, using an odd number of rules is enough to avoid tied scores. However, in multiclass problems this does not solve the issue. Score ties happens when two or more classes have the same highest score for a given sample, and can occur even with odd number of rules. It is recommended to use a sufficient number of rules in the model to reduce the chance of ties. Also, for class prediction and to minimize the tie chance `predict_one_vs_rest_TSP` function uses weighted class votes i.e., sum of the scores for the rules with value of true divided by sum of rule scores for that class instead of regular class votes i.e., number of rule with value of true divided by number of rules for that class. Ties are flagged and reported to the user and weighted votes is used by default to reduce the chance of ties.

```
# apply on the training data To have the classes in output in specific order, we
# can use 'classes' argument
results_train <- predict_one_vs_rest_TSP(classifier = classifier, Data = object,
    tolerate_missed_genes = TRUE, weighted_votes = TRUE)
```

```
## Get scores/votes from class:  LumB

## Get scores/votes from class:  Basal
```

```
## Get scores/votes from class:  LumA

## Get scores/votes from class:  Her2

## Get scores/votes from class:  Normal

## Checking the ties

## Score ties found in 42 out of 1500 samples in the data
```

```r
# apply on the test data weighted_votes is TRUE
results_test <- predict_one_vs_rest_TSP(classifier = classifier, Data = testing_data,
    tolerate_missed_genes = TRUE, weighted_votes = TRUE)
```

```
## Get scores/votes from class:  LumB

## Get scores/votes from class:  Basal

## Get scores/votes from class:  LumA

## Get scores/votes from class:  Her2

## Get scores/votes from class:  Normal

## Checking the ties

## Score ties found in 46 out of 1634 samples in the data
```

```r
# get a look over the scores in the test data
knitr::kable(head(results_test))
```

	LumB	Basal	LumA	Her2	Normal	max_score	tie_flag
F4	0.3023225	0	0.0407290	0.4648894	0.7356536	Normal	NA
F8	0.0000000	0	1.0000000	0.0589229	1.0000000	LumA	score_tie
F9	0.7294235	0	0.5177569	0.7695709	0.0691610	Her2	NA
F12	1.0000000	0	0.0000000	0.4141375	0.0000000	LumB	NA
F13	0.0000000	0	0.9607826	0.0573084	0.1997983	LumA	NA
F18	0.0000000	0	1.0000000	0.1182982	0.7310762	LumA	NA

We have score ties in 2.8 percent of the samples in the test data even with using `weighted_votes` option. This percentage may be reduced by using a higher number of rules in the model or by using the RF scheme which is less prone to tied scores.

By checking the accuracy in the training and test data, we got 88% overall accuracy in the training data and 84% overall accuracy in the test data. See the confusion matrices below:

```r
# Confusion Matrix and Statistics on training data
caret::confusionMatrix(data = factor(results_train$max_score, levels = unique(object$data$Labels)),
    reference = factor(object$data$Labels, levels = unique(object$data$Labels)))
```

```
## Confusion Matrix and Statistics
##
##           Reference
## Prediction LumB Basal LumA Her2 Normal
##     LumB    302     3   44   23      0
##     Basal     1   144    1    0      3
##     LumA     19     0  676    0     26
##     Her2      1     8   15  130      6
##     Normal    1     1   30    1     65
##
```

```
## Overall Statistics
##
##                Accuracy : 0.878
##                  95% CI : (0.8604, 0.8941)
##     No Information Rate : 0.5107
##     P-Value [Acc > NIR] : < 2.2e-16
##
##                   Kappa : 0.8193
##
##  Mcnemar's Test P-Value : 2.401e-09
##
## Statistics by Class:
##
##                      Class: LumB Class: Basal Class: LumA Class: Her2
## Sensitivity               0.9321      0.92308      0.8825     0.84416
## Specificity               0.9405      0.99628      0.9387     0.97771
## Pos Pred Value            0.8118      0.96644      0.9376     0.81250
## Neg Pred Value            0.9805      0.99112      0.8845     0.98209
## Prevalence                0.2160      0.10400      0.5107     0.10267
## Detection Rate            0.2013      0.09600      0.4507     0.08667
## Detection Prevalence      0.2480      0.09933      0.4807     0.10667
## Balanced Accuracy         0.9363      0.95968      0.9106     0.91093
##                      Class: Normal
## Sensitivity                0.65000
## Specificity                0.97643
## Pos Pred Value             0.66327
## Neg Pred Value             0.97504
## Prevalence                 0.06667
## Detection Rate             0.04333
## Detection Prevalence       0.06533
## Balanced Accuracy          0.81321
```

```r
# Confusion Matrix and Statistics on test data
caret::confusionMatrix(data = factor(results_test$max_score, levels = unique(object$data$Labels)),
    reference = factor(Test_Classes, levels = unique(object$data$Labels)))
```

```
## Confusion Matrix and Statistics
##
##           Reference
## Prediction LumB Basal LumA Her2 Normal
##     LumB    336     1   55   37      1
##     Basal     0   151    0    0      4
##     LumA     28     0  706    2     37
##     Her2      8    12   16  121      6
##     Normal    1     6   40    3     63
##
## Overall Statistics
##
##                Accuracy : 0.8427
##                  95% CI : (0.8241, 0.86)
##     No Information Rate : 0.5
##     P-Value [Acc > NIR] : < 2.2e-16
##
##                   Kappa : 0.7683
##
```

```
##  Mcnemar's Test P-Value : NA
##
## Statistics by Class:
##
##                      Class: LumB Class: Basal Class: LumA Class: Her2
## Sensitivity               0.9008      0.88824      0.8641     0.74233
## Specificity               0.9255      0.99727      0.9180     0.97145
## Pos Pred Value            0.7814      0.97419      0.9133     0.74233
## Neg Pred Value            0.9693      0.98715      0.8711     0.97145
## Prevalence                0.2283      0.10404      0.5000     0.09976
## Detection Rate            0.2056      0.09241      0.4321     0.07405
## Detection Prevalence      0.2632      0.09486      0.4731     0.09976
## Balanced Accuracy         0.9131      0.94275      0.8911     0.85689
##                      Class: Normal
## Sensitivity                0.56757
## Specificity                0.96717
## Pos Pred Value             0.55752
## Neg Pred Value             0.96844
## Prevalence                 0.06793
## Detection Rate             0.03856
## Detection Prevalence       0.06916
## Balanced Accuracy          0.76737
```

4.4 Visualization

Finally it is recommended to visualize the rules in the training and test data to examine rule and prediction behavior and quality. We can plot binary heatmap plots through `plot_binary_TSP` function as follows:

```
# plot for the rules and scores in the training data
plot_binary_TSP(Data = object, # we are using the data object here
                classifier = classifier,
                prediction = results_train,
                show_rule_name = FALSE,
                title = "Training data")

# plot for the rules and scores in the test data
plot_binary_TSP(Data = testing_data,
                ref = Test_Classes,
                classifier = classifier,
                prediction = results_test,
                show_rule_name = FALSE,
                title = "Test data")
```

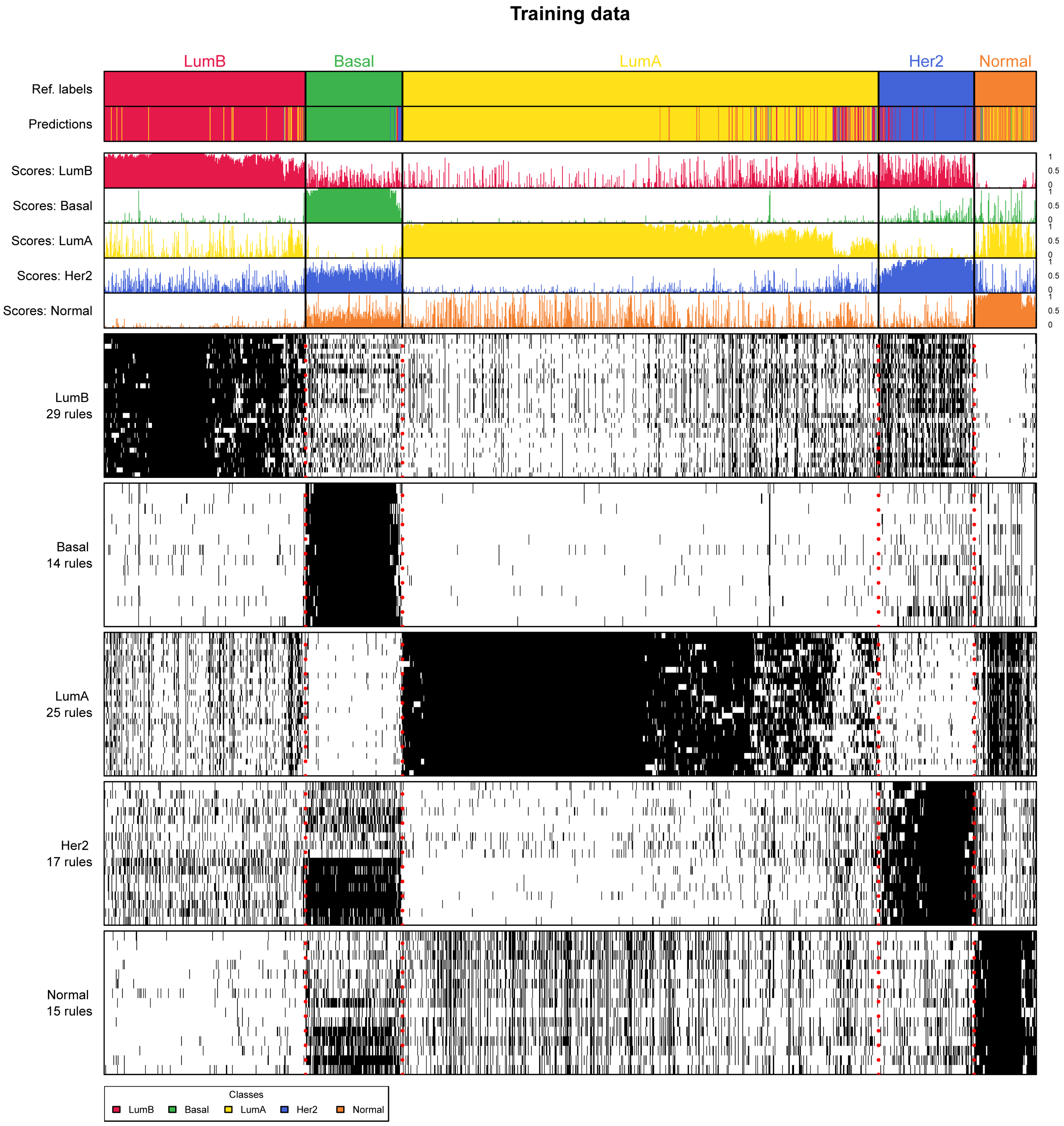

Training data
LumB
Basal
LumA
Her2
Normal
Ref. labels
Predictions
Scores: LumB
Scores: Basal
Scores: LumA
Scores: Her2
Scores: Normal
1
0.5
0
LumB
29 rules
Basal
14 rules
LumA
25 rules
Her2
17 rules
Normal
15 rules
Classes
LumB
Basal
LumA
Her2
Normal

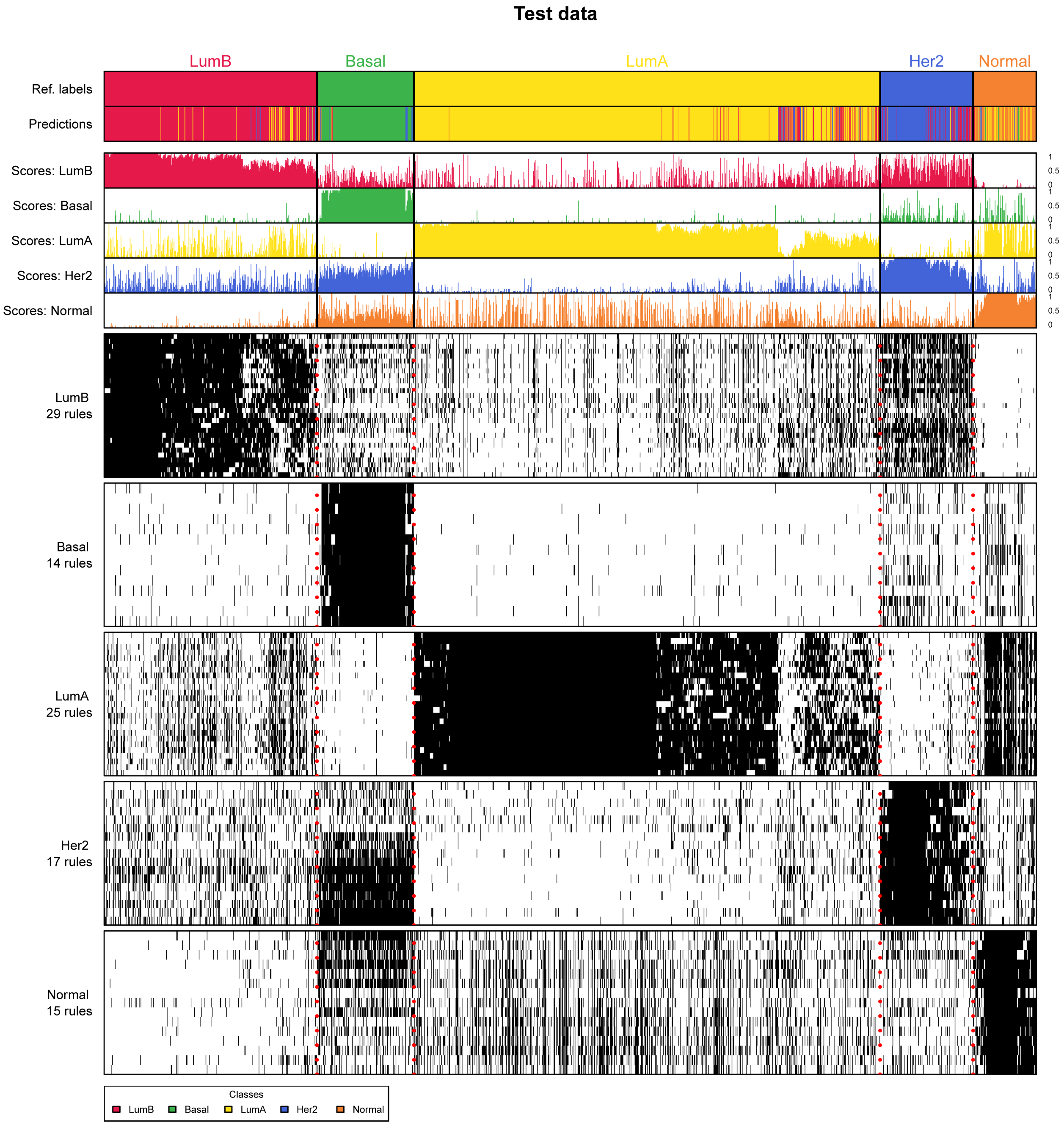
Test data
LumB
Basal
LumA
Her2
Normal
Ref. labels
Predictions
Scores: LumB
Scores: Basal
Scores: LumA
Scores: Her2
Scores: Normal
1
0.5
0
LumB
29 rules
Basal
14 rules
LumA
25 rules
Her2
17 rules
Normal
15 rules
Classes
LumB
Basal
LumA
Her2
Normal

5 Random Forest scheme

In the Random Forest (RF) scheme, all steps of gene filtering/sorting, rule filtering/sorting, and final model training are performed using the RF algorithm. The RF approach trades some of the intuitive interpretability of the one-vs-rest model but often gains an advantage in accuracy and scoring approach (less ties).

The `ranger` package is used to run the RF algorithm, and arguments can be passed to `ranger` function if any parameter tuning is needed. For example, increasing the number of trees or modifying the `mtry` (e.g., using 10% of variables instead of the square root of the number of variables).

5.1 Gene sorting

The `sort_genes_RF` function sorts the genes based on their importance. Gene importance is determined based on the ability of the gene to split the classes better than other genes as described in `ranger` package.

To get the important (i.e. informative) genes, the `sort_genes_RF` function performs two types of gene sorting, the first type is "altogether" which runs the RF algorithm to sort the genes based on their importance in all classes from each other, and this generates one list of sorted genes for the whole data. The second type is "one_vs_rest" which runs the RF algorithm to sort the genes based on their importance in splitting one class from the rest, and this generates sorted list of genes for each class. It is recommended (default) to run both, particularly in case of class imbalance in the training data.

The number of returned genes can be specified using `featureNo_altogether` and `featureNo_one_vs_rest` arguments. If one of these arguments is set to 0, that sorting type will be skipped and not return any genes. By default, all genes are sorted and returned through both sorting types, and the user specifies how many top genes will be used in the downstream functions (`sort_rules_RF`).

Like the one-vs-rest scheme, platform-wise option is available for the RF scheme where the genes and rules are sorted in each platform/study separately, then the top genes in all of them will be taken. This gives more weight to small platforms/studies and try to get genes that function equally well in all platforms/studies.

Note that the data is not ranked by default, but you can rank the data (ex. in case you are using non-normalized data) before sorting the genes using `rank_data argument`, the genes then will be ranked within each sample.

Data can be ranked (e.g. if non-normalized data is used) before sorting the genes through the `rank_data` argument. The genes will be ranked within each sample separately.

Running the gene sorting function on the training data object:

```
genes_RF <- sort_genes_RF(data_object = object, platform_wise = FALSE, seed = 123456)
```

```
genes_RF  # sorted genes object
```

```
## multiclassPairs - Random Forest scheme
##   Object contains:
##       - sorted_genes
##          - class: all : 18213 genes
##          - class: LumB : 18213 genes
##          - class: Basal : 18213 genes
##          - class: LumA : 18213 genes
##          - class: Her2 : 18213 genes
##          - class: Normal : 18213 genes
##       - RF_classifiers
##       - calls
##             sort_genes_RF(data_object = object,
##              rank_data = F,
##              seed = 123456)
```

5.2 Rule sorting

After we sorted the genes, we need to take the top genes and combine them as binary rules and then sort these rules. This is performed using the `sort_rules_RF` function. Here, we need to specify how many top genes to use.

Like the gene sorting options, rules sorting can be performed in "altogether", "one_vs_rest", and with a "platform_wise" option. By default, both "altogether" and "one_vs_rest" rule sorting are performed.

```
# Run sort_rules_RF to create the rules and sort them
rules_RF <- sort_rules_RF(data_object = object, sorted_genes_RF = genes_RF, genes_altogether = 50,
    genes_one_vs_rest = 50, seed = 123456)
```

```
rules_RF  # sorted rules object
```

```
## multiclassPairs - Random Forest scheme
##   Object contains:
##       - sorted_genes
##          - class: all : 50 genes
##          - class: LumB : 50 genes
##          - class: Basal : 50 genes
##          - class: LumA : 50 genes
##          - class: Her2 : 50 genes
##          - class: Normal : 50 genes
##       - sorted_rules
##          - class: all : 28441  sorted rules
##          - class: LumB : 28441  sorted rules
##          - class: Basal : 28441  sorted rules
##          - class: LumA : 28441  sorted rules
##          - class: Her2 : 28441  sorted rules
##          - class: Normal : 28441  sorted rules
##       - RF_classifiers
##       - calls
##             sort_rules_RF(data_object = object,
##              sorted_genes_RF = sorted_genes,
##              genes_altogether = 50,
##              genes_one_vs_rest = 50,
##              seed = 123456)
```

Optional helper function to the `sort_rules_RF` function:

The optional `summary_genes_RF` function can be used before the running the `sort_rules_RF` step to calculate how many unique genes and rules that a specified `genes_altogether` and `genes_one_vs_rest` would generate. This can help the user determine a suitable setting for these parameters.

The next chunk show an example for `summary_genes_RF` function:

```
summary_genes_RF(sorted_genes_RF = genes_RF, genes_altogether = c(20, 50, 100, 200),
    genes_one_vs_rest = c(20, 50, 100, 200))
```

genes altogether in object	genes 1 vs r in object	n classes	from altogether	from one vs rest	n unique genes	n rules
18213	18213	5	20	20	98	4753
18213	18213	5	50	50	239	28441
18213	18213	5	100	100	457	104196
18213	18213	5	200	200	855	365085

5.3 Model training

With the rules sorted based on their importance. We can now use the `train_RF` function to train our final rule-based RF classifier.

The model can be trained using the argument `run_boruta=TRUE`, to remove any potentially uninformative rules remaining in the model through `Boruta` package (Kursa and Rudnicki, 2010). This can often reduce the number of used genes and rules in the final model, without impacting prediction performance. Using `run_boruta=TRUE` increases the training time.

By default, the final classifier is trained with `probability=TRUE`, allowing the model to return class prediction scores rather than only the class prediction.

```
# train the final model
RF_classifier <- train_RF(data_object = object, sorted_rules_RF = rules_RF, rules_altogether = 50,
    rules_one_vs_rest = 50, gene_repetition = 1, run_boruta = TRUE)
```

```
RF_classifier
```

```
## multiclassPairs - Rule based Random Forest classifier
##   Object contains:
##       - genes: 212 genes
##       - rules: 263 rules
##       - TrainingMatrix      - boruta
##       - RF_classifier
##       - calls: train_RF(data_object = object,
##              sorted_rules_RF = sorted_rules,
##              rules_altogether = 50,
##              rules_one_vs_rest = 50,
##              run_boruta = T,
##              seed = 123456)
```

Optional parameter optimization:

To evaluate the performance of different parameter settings we can use the `optimize_RF` function to make a grid search with user-specified parameters as demonstrated in the next code chunk:

```
# prepare the simple data.frame for the parameters we want to test
# names of arguments as column names
parameters <- expand.grid(
  gene_repetition=c(3,1),
  rules_one_vs_rest=c(10,30,50),
  rules_altogether=c(10,30,50),
  run_boruta=FALSE,
  stringsAsFactors = FALSE)

# for overall and byclass possible options, check the help files
para_opt <- optimize_RF(data_object = object,
                        sorted_rules_RF = rules_RF,
                        parameters = parameters,
                        test_object = NULL,
                        overall = c("Accuracy","Kappa"), # overall measurements
                        byclass = c("F1"), # measurements per class
                        verbose = TRUE)
# para_opt
# para_opt$summary # the df of with summarized information
```

5.4 Training Accuracy

For the training accuracy, we do not apply the RF model on the training data, because this gives 100% accuracy (almost always) which is not reliable. Instead we use the out-of-bag predictions, which can be obtained as follows:

```
# training accuracy get the prediction labels from the trained model if the
# classifier trained using probability = FALSE
training_pred <- RF_classifier$RF_scheme$RF_classifier$predictions
if (is.factor(training_pred)) {
    x <- as.character(training_pred)
}
# if the classifier trained using probability = TRUE
if (is.matrix(training_pred)) {
    x <- colnames(training_pred)[max.col(training_pred)]
}
# training accuracy
caret::confusionMatrix(data = factor(x), reference = factor(object$data$Labels))
```

```
## Confusion Matrix and Statistics
##
##           Reference
## Prediction Basal Her2 LumA LumB Normal
##     Basal    145    1    0    2      2
##     Her2       3  125    1    3      2
##     LumA       5    7  736   32     34
##     LumB       2   19   24  287      1
##     Normal     1    2    5    0     61
##
## Overall Statistics
##
##                Accuracy : 0.9027
##                  95% CI : (0.8865, 0.9172)
##     No Information Rate : 0.5107
##     P-Value [Acc > NIR] : < 2.2e-16
##
##                   Kappa : 0.8508
##
##  Mcnemar's Test P-Value : 1.332e-06
##
## Statistics by Class:
##
##                      Class: Basal Class: Her2 Class: LumA Class: LumB
## Sensitivity               0.92949     0.81169      0.9608      0.8858
## Specificity               0.99628     0.99331      0.8937      0.9609
## Pos Pred Value            0.96667     0.93284      0.9042      0.8619
## Neg Pred Value            0.99185     0.97877      0.9563      0.9683
## Prevalence                0.10400     0.10267      0.5107      0.2160
## Detection Rate            0.09667     0.08333      0.4907      0.1913
## Detection Prevalence      0.10000     0.08933      0.5427      0.2220
## Balanced Accuracy         0.96288     0.90250      0.9273      0.9233
##                      Class: Normal
## Sensitivity                0.61000
## Specificity                0.99429
## Pos Pred Value             0.88406
```

```
## Neg Pred Value              0.97275
## Prevalence                  0.06667
## Detection Rate              0.04067
## Detection Prevalence        0.04600
## Balanced Accuracy           0.80214
```

5.5 Prediction

Prediction is performed by running the `predict_RF` function. The `impute = TRUE` argument can be used to handle missed genes in the test data. This is performed by the K-nearest neighbor (kNN) imputation of missing rule values based on the closest samples in the training rule data (saved within the model).

We got 90% overall accuracy in the training data and 88% overall accuracy in the test data as shown in the confusion matrix below.

```r
# apply on test data
results <- predict_RF(classifier = RF_classifier, Data = testing_data, impute = TRUE,
    verbose = TRUE)
# we did not get any score tie warnings

# training accuracy
caret::confusionMatrix(data = factor(results$predictions_classes), reference = factor(Test_Classes))
```

```
## Confusion Matrix and Statistics
##
##             Reference
## Prediction Basal Her2 LumA LumB Normal
##     Basal    154    2    0    0      0
##     Her2       7  134    5    7      4
##     LumA       4    9  777   48     44
##     LumB       2   17   22  318      0
##     Normal     3    1   13    0     63
##
## Overall Statistics
##
##                Accuracy : 0.8849
##                  95% CI : (0.8685, 0.9)
##     No Information Rate : 0.5
##     P-Value [Acc > NIR] : < 2.2e-16
##
##                   Kappa : 0.8249
##
##  Mcnemar's Test P-Value : NA
##
## Statistics by Class:
##
##                      Class: Basal Class: Her2 Class: LumA Class: LumB
## Sensitivity               0.90588     0.82209      0.9510      0.8525
## Specificity               0.99863     0.98436      0.8715      0.9675
## Pos Pred Value            0.98718     0.85350      0.8810      0.8858
## Neg Pred Value            0.98917     0.98037      0.9468      0.9569
## Prevalence                0.10404     0.09976      0.5000      0.2283
## Detection Rate            0.09425     0.08201      0.4755      0.1946
## Detection Prevalence      0.09547     0.09608      0.5398      0.2197
## Balanced Accuracy         0.95226     0.90323      0.9113      0.9100
```

```
##                      Class: Normal
## Sensitivity                0.56757
## Specificity                0.98884
## Pos Pred Value             0.78750
## Neg Pred Value             0.96911
## Prevalence                 0.06793
## Detection Rate             0.03856
## Detection Prevalence       0.04896
## Balanced Accuracy          0.77820
```

5.6 Visualization

plot_binary_RF can be used to plot binary heatmaps for the rules in training and test datasets, as follow:

```
#visualize the binary rules in training dataset
plot_binary_RF(Data = object,
               classifier = RF_classifier,
               as_training = TRUE, # to extract the scores from the model
               top_anno = "ref",
               show_rule_name = FALSE,
               title = "Training data")
```

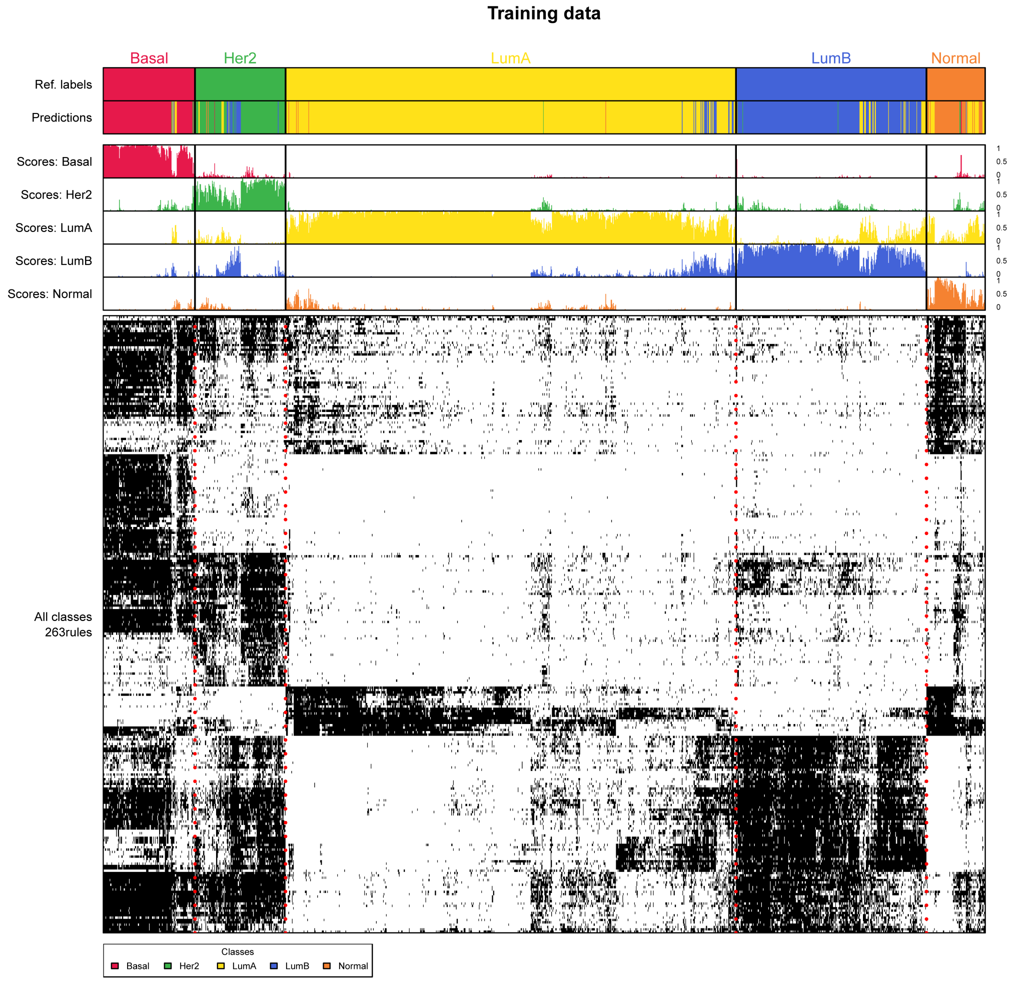

```
# visualize the binary rules in test dataset
plot_binary_RF(Data = testing_data, ref = Test_Classes, classifier = RF_classifier,
    prediction = results, as_training = FALSE, top_anno = "ref", show_rule_name = FALSE,
    title = "Test data")
```

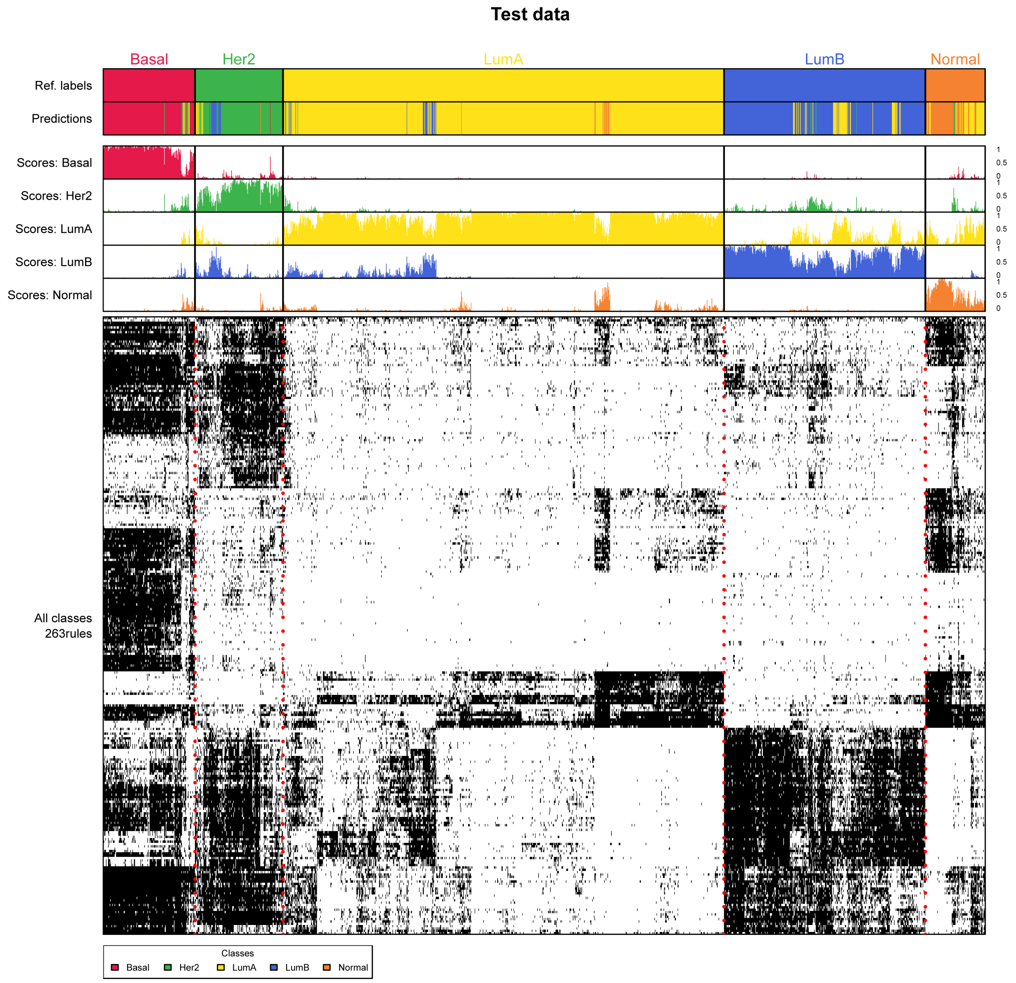

Training data - quality check:

From the trained model we can extract a proximity matrix which is based on the fraction of times any given out-of-bag samples receive the same predicted label in each tree. This can give an overview of the predictor performance in the training data, and the behavior and cohesiveness of our reference labels. We can generate a heatmap for the proximity matrix by `proximity_matrix_RF` function as follows:

```
# plot proximity matrix of the out-of-bag samples
# Note: this takes a lot of time if the data is big
proximity_matrix_RF(object = object,
                    classifier = RF_classifier,
                    plot = TRUE,
                    return_matrix = FALSE, # if we want to extract and save the matrix
                    title = "Breast Cancer",
                    cluster_cols = TRUE)
```

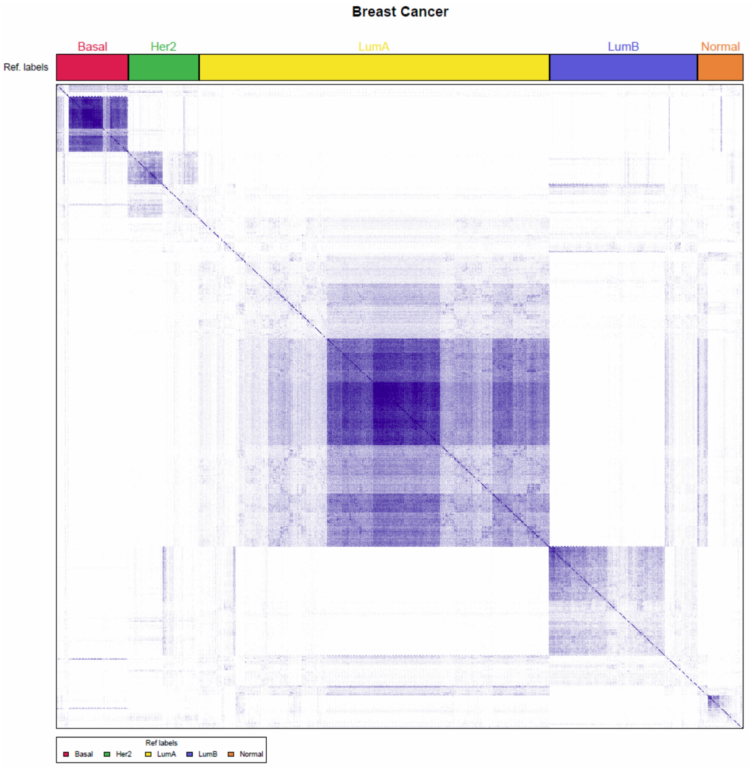

6 Disjoint rules

`multiclassPairs` allows the user to select if the rules should be disjoint or not in the final model. This means if the gene is allowed to be repeated in the rules or not. For the one-vs-rest scheme, we pass the `disjoint` argument to switchBox package to allow/prevent the gene repetition in the rules. In the random forest scheme, we give the user more control over the gene repetition where the `gene_repetition` argument in the `train_RF` function allows the user to determine how many times the gene is allowed to be repeated in the rules. When `gene_repetition` = 1 the produced classifier will have disjointed rules.

7 Comparison

Here, we compare the runtime and accuracy of the two schemes in `multiclassPairs` (one-vs-rest and random forest) with the multiclass pair-based decision tree (DT) implementation in `Rgtsp` package (Popovici et al.,

2011).

We used the same breast cancer training data as in the previous sections and ran the three approaches subsetting different training cohort sizes: 50, 100, 200, 500, 1000, and 1500 samples. The default settings for all approaches were used with minor changes as in the next chunk. We repeated the training process 5 times and recorded the training time for the full pipeline (i.e. gene and rule filtering and model training) and the accuracy. We used two different computational settings: 4 cores/64GB of RAMs and 24 cores/128GB of RAMs. The same test data as in the previous sections was used (n=1634 samples).

We found that the DT models had the lowest accuracy and the highest training time regardless of the training dataset size (Figure 5). RF models with training sample sizes above 50 outperformed both one-vs-rest and the DT models (Figure 5). One-vs-rest models showed the lowest training time with the small training datasets, while RF approach showed the lowest training time in the model with 1500 training samples (Figure 6).

```
# For one-vs-rest scheme settings:
# one_vs_rest gene filtering with k_range of 10:50
object <- ReadData(Data = training_data,
                   Labels = Train_Classes)

filtered_genes <- filter_genes_TSP(data_object = object,
                                   featureNo = 1000, UpDown = TRUE,
                                   filter = "one_vs_rest")

classifier_1_vs_r <- train_one_vs_rest_TSP(data_object = object,
                                           filtered_genes = filtered_genes,
                                           k_range = 10:50, disjoint = TRUE,
                                           include_pivot = FALSE,
                                           platform_wise_scores = FALSE,
                                           one_vs_one_scores = FALSE)

# For RF scheme settings:
# Settings represents a large model to show calculation time with high number of genes/rules
object <- ReadData(Data = training_data,
                   Labels = Train_Classes)

sorted_genes  <- sort_genes_RF(object)

sorted_rules  <- sort_rules_RF(data_object = object,
                               sorted_genes_RF = sorted_genes,
                               genes_altogether = 200,
                               genes_one_vs_rest = 200)

classifier_RF <- train_RF(data_object = object,
                          sorted_rules_RF = sorted_rules,
                          rules_one_vs_rest = 200,
                          rules_altogether = 200,
                          run_boruta = FALSE, gene_repetition = 1)

# For DT scheme from Rgtsp package:
# default settings with min.score=0.6 (to avoid running out of rules with the default setting 0.75)
classifier_DT <- mtsp(X = t(as.matrix(training_data)),
                      y = Train_Classes,
                      min.score=0.60)
```

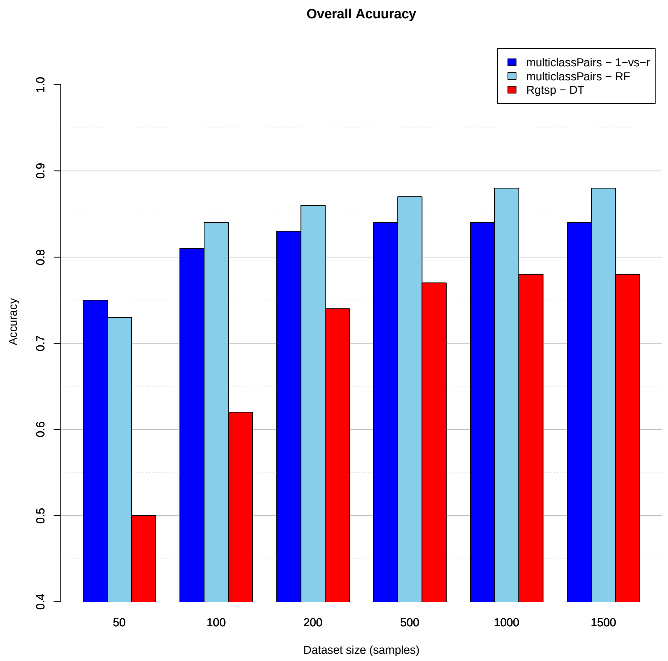

Figure 5: Overall accuracy for the three pair-based approaches. X-axis show the performance of different models trained on training datasets with different sizes.

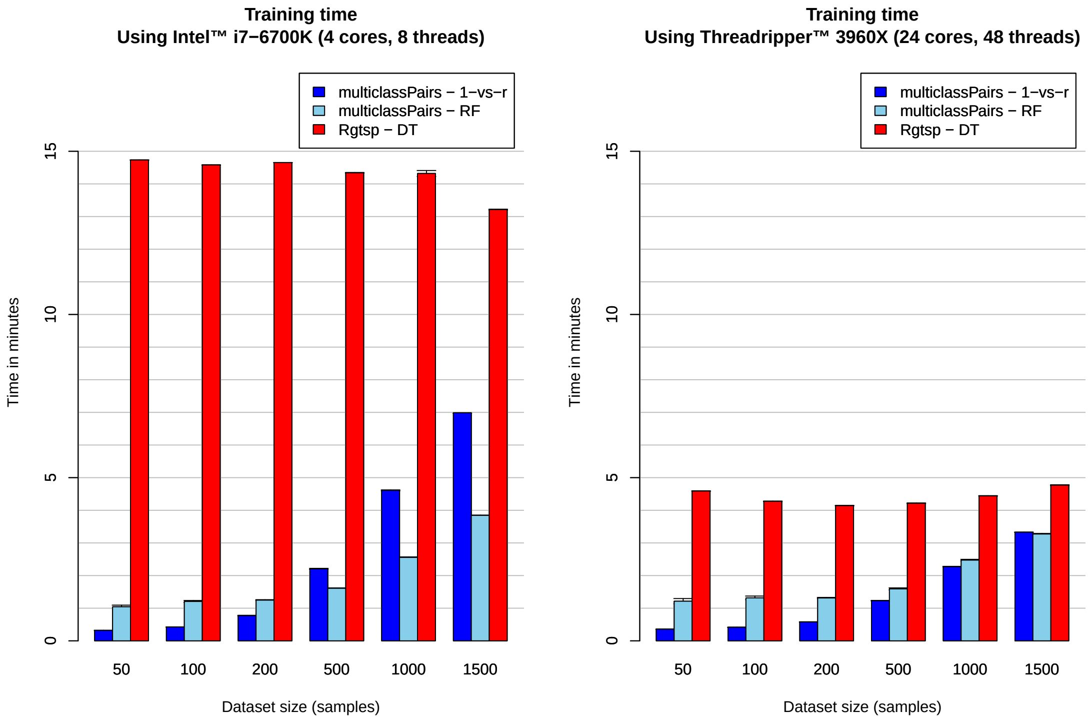

Figure 6: Average of the overall training time including the gene and rules filtering and model training. Training repeated 5 times for each model and the bars show the average time.

8 References

Afsari, B., Braga-Neto, U.M., and Geman, D. (2014). Rank discriminants for predicting phenotypes from RNA expression. Ann. Appl. Stat. 8, 1469–1491.

Afsari, B., Fertig, E.J., Geman, D., and Marchionni, L. (2015). switchBox: an R package for k-Top Scoring Pairs classifier development. Bioinforma. Oxf. Engl. 31, 273–274.

Brueffer, C., Vallon-Christersson, J., Grabau, D., Ehinger, A., Häkkinen, J., Hegardt, C., Malina, J., Chen, Y., Bendahl, P.-O., Manjer, J., et al. (2018). Clinical Value of RNA Sequencing-Based Classifiers for Prediction of the Five Conventional Breast Cancer Biomarkers: A Report From the Population-Based Multicenter Sweden Cancerome Analysis Network-Breast Initiative. JCO Precis. Oncol. 2.

Kursa, M.B., and Rudnicki, W.R. (2010). Feature Selection with the Boruta Package. J. Stat. Softw. 36, 1–13.

Popovici, V., Budinská, E., and Delorenzi, M. (2011). Rgtsp: a generalized top scoring pairs package for class prediction. Bioinforma. Oxf. Engl. 27, 1729–1730.

Tan, A.C., Naiman, D.Q., Xu, L., Winslow, R.L., and Geman, D. (2005). Simple decision rules for classifying human cancers from gene expression profiles. Bioinforma. Oxf. Engl. 21, 3896–3904.

Wright, M.N., and Ziegler, A. (2017). ranger: A Fast Implementation of Random Forests for High Dimensional Data in C++ and R. J. Stat. Softw. 77, 1–17.

9 Session info

```
sessionInfo()
```

```
## R version 4.0.3 (2020-10-10)
## Platform: x86_64-w64-mingw32/x64 (64-bit)
## Running under: Windows 10 x64 (build 19042)
##
## Matrix products: default
##
## locale:
## [1] LC_COLLATE=English_United States.1252
## [2] LC_CTYPE=English_United States.1252
## [3] LC_MONETARY=English_United States.1252
## [4] LC_NUMERIC=C
## [5] LC_TIME=English_United States.1252
##
## attached base packages:
## [1] stats     graphics  grDevices utils     datasets  methods   base
##
## other attached packages:
## [1] multiclassPairs_0.4.1 knitr_1.30            BiocStyle_2.16.1
##
## loaded via a namespace (and not attached):
##  [1] Rcpp_1.0.6           lubridate_1.7.9.2    lattice_0.20-41
##  [4] class_7.3-17         assertthat_0.2.1     digest_0.6.27
##  [7] ipred_0.9-9          foreach_1.5.1        R6_2.5.0
## [10] ranger_0.12.1        plyr_1.8.6           stats4_4.0.3
## [13] e1071_1.7-4          evaluate_0.14        ggplot2_3.3.3
## [16] pillar_1.4.7         rlang_0.4.10         caret_6.0-86
## [19] rstudioapi_0.13      data.table_1.13.6    rpart_4.1-15
## [22] Matrix_1.3-2         rmarkdown_2.6        splines_4.0.3
## [25] gower_0.2.2          stringr_1.4.0        munsell_0.5.0
## [28] compiler_4.0.3       xfun_0.20            pkgconfig_2.0.3
## [31] Boruta_7.0.0         htmltools_0.5.1.1    nnet_7.3-14
## [34] tidyselect_1.1.0     tibble_3.0.5         prodlim_2019.11.13
## [37] codetools_0.2-18     dunn.test_1.3.5      crayon_1.3.4
## [40] dplyr_1.0.3          withr_2.4.0          MASS_7.3-53
## [43] recipes_0.1.15       ModelMetrics_1.2.2.2 grid_4.0.3
## [46] nlme_3.1-151         gtable_0.3.0         lifecycle_0.2.0
## [49] DBI_1.1.1            formatR_1.7          magrittr_2.0.1
## [52] pROC_1.17.0.1        scales_1.1.1         stringi_1.5.3
## [55] reshape2_1.4.4       timeDate_3043.102    ellipsis_0.3.1
## [58] generics_0.1.0       vctrs_0.3.6          rdist_0.0.5
## [61] lava_1.6.8.1         iterators_1.0.13     tools_4.0.3
## [64] glue_1.4.2           purrr_0.3.4          survival_3.2-7
## [67] yaml_2.2.1           colorspace_2.0-0     BiocManager_1.30.10
```
